# Peri‐ankle muscles architecture and performance changes in patients with chronic ankle instability: A retrospective cross‐sectional study

**DOI:** 10.1002/jfa2.12035

**Published:** 2024-07-06

**Authors:** Heeju Yu, Seungmi Yeo, Ji Young Lim, Inah Kim, Jihye Hwang, Wan‐hee Lee

**Affiliations:** ^1^ Department of Physical Therapy Sahmyook University College of Health Science Seoul Republic of Korea; ^2^ Department of Rehabilitation Medicine Pusan National University Yangsan Hospital Pusan National University School of Medicine Yangsan Republic of Korea; ^3^ Department of Physical and Rehabilitation Medicine Medical Research Institute Sungkyunkwan University School of Medicine Suwon Republic of Korea; ^4^ Department of Physical and Rehabilitation Medicine Dongtan Sacred Heart Hospital Hallym University College of Medicine Hwaseong Republic of Korea; ^5^ Department of Physical and Rehabilitation Medicine Samsung Medical Center Sungkyunkwan University School of Medicine Seoul Republic of Korea

**Keywords:** chronic ankle instability, muscle thickness, pennation angle, ultrasound

## Abstract

This study aimed to identify changes in the architecture and performance of the peri‐ankle muscles in patients with chronic ankle instability (CAI) and investigate the relationship between them. In total, 17 subjects were evaluated retrospectively. Each subject underwent anthropometric and isokinetic test, and peroneus longus (PL) and brevis (PB), medial gastrocnemius (MGCM), and tibialis anterior (TA) ultrasound imaging were performed at rest and maximum voluntary contraction (MVC) conditions. Regarding muscle architectural variables, the pennation angle (PA) of the MGCM at rest and the PA of the TA, MGCM, and PL in MVC were significantly reduced on the injured side compared to the intact side. There were no significant differences in muscle thickness of PL, PB, MGCM, and TA observed between intact and injured side during both rest and MVC. Regarding muscle performance parameters, significant decreased were observed in the muscle strength for both limbs in all four directions under the two different conditions. A secondary finding was that the relative PA ratio of the TA showed moderate correlation with the relative dorsiflexion ratio at 30°/s. These findings can provide opportunities to better understand how injuries in patients with CAI may be related to changes in ankle and foot function.

## 1 INTRODUCTIONS

Ankle sprains are among the most frequent musculoskeletal injuries that occur during sports, recreation, or other physical activities [1]. A high percentage of ankle sprains are lateral ankle sprains, which are typically caused by the excessive supination of the hindfoot on an externally rotated tibia [2]. Approximately 40% of the individuals who have experienced an initial ankle sprain develop chronic ankle instability (CAI) [3], which presents with persistent pain, weakness, and instability, both mechanically and functionally [4].

While the mechanisms behind the development of CAI are not fully understood, researchers have observed reduced muscle activation [5], prolonged muscle response to perturbation [6], and altered onset of muscle activity of the evertor, plantar flexor, and dorsiflexor after ankle sprains [7, 8]. Kim (2019) showed that ankle sprains can lead to decreased activation and coordination of three critical peri‐ankle muscles (peroneus longus (PL), gastrocnemius (GCM), and tibialis anterior (TA)) during functional activities, potentially increasing the risk of recurrent ankle sprains in the future [9]. Mansfield and Neumann suggested that weakness in the peroneus muscles can make the foot more susceptible to inversion, which is a common factor of lateral ankle sprains [10].

Given that the architecture of weaknesses or imbalances in these muscles has a strong impact on altered ankle kinematics, understanding the changes of architectures and performance of peri‐ankle muscles, which provide stability to the ankle and foot, will be helpful in reducing the risk of further injury [11, 12, 13]. However, as far as we know, there has not been a comprehensive study into the architectural and performance changes in the peri‐ankle muscles of patients with CAI, and how these changes are correlated.

The primary objective of this study was to identify changes in the architecture and performance of the peri‐ankle muscles (PL, PB, medial gastrocnemius (MGCM), and TA) of patients with CAI, by comparing both the injured and intact side. The secondary objective of the study was to investigate the correlation between the architecturally altered muscles and their performance, in comparison to the intact side.

## 2 METHODS

### 2.1 Study design and subjects

In this retrospective cross‐sectional study, patients with CAI who visited a tertiary care hospital in Seoul, Republic of Korea from September 2020 to October 2022, were selected as the subjects of the research. We first reviewed the cross‐sectional baseline data from the electronic medical records (EMR), discarding entries with incomplete information, inconsistent data, and poor‐quality ultrasound imaging. This process resulted in 22 medical records. Subsequently, we filtered the dataset by applying the inclusion criteria established by the International Ankle Consortium to identify patients with CAI [14]. Following screening, 17 medical records met the inclusion criteria and were subsequently analyzed for conducting the retrospective study.

The inclusion criteria of this study, which were as follows: (1) subjects aged 18 years or older; (2) a previous history of at least one severe ankle sprain that caused pain, swelling, limited weight bearing, or complete immobility for at least three days; (3) failure to return to pre‐injury fxunctionality; (4) repeated episodes of ankle sprain; and (5) a self‐reported ankle dysfunction score of ≤24 on the Cumberland Ankle Instability Tool (CAIT) [15], classified as a noticeable or pathologic condition [16]. The exclusion criteria were as follows: (1) experienced an injury within the 3 months prior to the first outpatient visit; (2) history of surgery on their bones, joint structures, or nerves in either of their lower limbs; (3) previously experienced a fracture in either of their lower limbs; (4) acute injury to the musculoskeletal structures in their lower extremities within the previous 3 months that impacted their joint function and resulted in at least one day of interrupted physical activity; (5) experienced current and/or intermittent pain; and (6) systemic musculoskeletal disease.

## 3 DATA ACQUISITION

Data collected from the EMR included sex, age, body mass index, affected side, dominant side, CAIT score, and time since the first sprain. The clinical variables included peri‐ankle muscle architecture and performance. The architectural variables of the peri‐ankle muscles, specifically the pennation angle (PA) and muscle thickness (MT), were obtained using ultrasound imaging. Muscle performance variables were assessed by whole leg isokinetic test, measuring peak torque during ankle eversion (AE), ankle inversion (AI), ankle plantar flexion (AP), and ankle dorsiflexion (AD), and measured at two different velocities (30°/s and 120°/s).

### 3.1 Muscle architectural variables

The muscle architectural variables were measured using an HM70A (Samsung Medicine, Gangwon‐do, Korea) or Volusion E6 system (GE Healthcare), both equipped with a 5–13 MHz linear transducer. The PA and MT of peri‐ankle muscles were scanned once at rest and once during maximum voluntary contraction (MVC) conditions using B‐mode (brightness mode). For all tests were obtained under two conditions (resting and MVC). A musculoskeletal physiatrist experienced in ultrasound carried out the ultrasound scans of the subjects, and a physical therapist was responsible for fixing the subjects' initial starting position and providing resistance during the MVC of each muscle.

#### 3.1.1 Peroneus muscles

**FIGURE 1:**
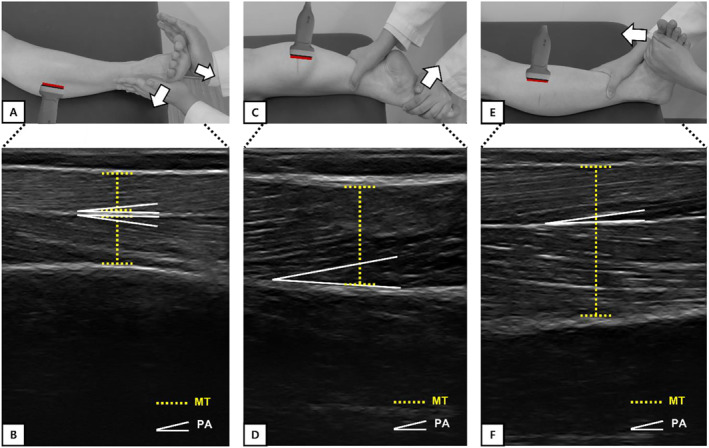
(A) Assessment of peroneal muscles at MVC; (B) ultrasound image of the peroneal muscles; (C) assessment of MGCM at MVC; (D) ultrasound image of the MGCM; (E) assessment of TA at MVC; (F) ultrasound image of the TA. MGCM, medial gastrocnemius; TA, tibialis anterior.

The PA and MT of the peroneal muscles were evaluated separately as PL and PB. The probe was placed at a location equivalent to 30% of the distance between the lateral malleolus and the fibular head relative to the head of the fibula in the supine position [17]. Next, the probe was positioned in the longitudinal direction to obtain measurements for PA and MT. Images of the peroneus muscles were obtained under two conditions: (1) with the ankle joint positioned at 90° (neutral position) while the subject was at rest, and (2) during MVC by providing resistance to the main concentric actions of target muscles (AE and AP) (Figure 1A) [18]. PA and MT were analyzed between the side and central apophysis (see Figure 1) [17].

#### 3.1.2 Medial gastrocnemius

To assess the MGCM, the subject lay prone with their feet hanging off the edge of the table and knees completely extended. The PA and MT of the MGCM were scanned at the same distance as those of the peroneus muscles, and the probe was positioned longitudinally and moved medially to the MGCM [17]. Images of the MGCM were obtained under two conditions: (1) with the ankle joint positioned at 90° (neutral position) while the subject was at rest, and (2) during MVC by providing resistance to the main concentric action of target muscle (AP) (Figure 1C). The PA was defined as the insertion angle of the muscle fascicle into the deep aponeurosis [19] and the MT of the MGCM was determined as the distance between the superficial and deep aponeuroses at the center of the image (see Figure 1) [17].

#### 3.1.3 Tibialis anterior

The PA and MT in the TA were measured at the same distance as the peroneus muscles on a bed in the supine position. The probe was placed lengthwise, and the image was taken when the TA reached its maximum achievable endpoint line [17]. Images of the TA were obtained under two conditions: (1) with the ankle joint positioned at 90° (neutral position) while the subject was at rest, and (2) during MVC by providing resistance to the main concentric action of target muscle (AD) (Figure 1E) [20]. The PA of the TA was analyzed as the angle of insertion of the fascicles into the central aponeurosis of the muscle, and the MT was determined as the distance between the superficial and deep aponeuroses of the muscle fibers in the middle of the image (see Figure 1) [21].

### 3.2 Muscle performance variables

The study analyzed peak torque which measures dynamic muscle function [22] as a muscle performance variable for four movement tasks (AE, AI, AP, and AD) at two different angular velocities (30°/s and 120°/s) during concentric muscle contractions using an isokinetic dynamometer (Biodex System 3, Biodex Medical Systems, Shirley, USA). All of the peak torque values were normalized to each subject's body mass (Nm/kg). Moreover, the relative deficit of the involved side was used for statistical analysis calculated as follows [23, 24]: (intact side–injured side)/intact side × 100%.

## 4 STATISTICAL ANALYSIS

All statistical analyses were performed using SPSS statistical software (version 29.0; IBM). Mean values with standard deviation (SD) were used to report continuous variables, while categorical variables were presented as numbers and ratios. The Shapiro–Wilk test was conducted to confirm the normal distribution of the data. To compare clinical variables between the injured and intact side, the paired sample *t*‐test was used. Pearson's correlation analysis was employed to evaluate the association between PA (ratio of injured side to intact side under MVC conditions) among the ultrasound parameters and normalized isokinetic peak torque ratio of the injured to intact side. The Pearson correlation coefficient (*r*) was interpreted as a value: 0.10–0.39 classified as a weak correlation, 0.40–0.69 as a moderate correlation, 0.70–0.89 as a strong correlation, and 0.90 or larger as a very strong correlation [25]. All statistical significance levels were set as 0.05.

## 5 RESULTS

Patients' characteristics of the 17 included participants are displayed in Table 1. Twelve subjects (70.59%) were female. The mean age of the 17 subjects was 33.59 (SD, 11.84) years. Nine subjects (52.94%) were affected with dominant extremity. From the time since the first sprain, more than one year had passed for 11 subjects (64.71%), while the remaining subjects (35.29%) had experienced their first sprain more than three months ago but less than a year ago. The mean CAIT scores of the injured side were 8.06 ± 5.26 and 26.12 ± 4.76 for the intact side.

**TABLE 1:** Subjects' characteristics (*N* = 17).

Variables	Mean ± SD or *n* (%)
Sex, female (%)	12 (70.59)
Age (years)	33.59 ± 11.84
Body mass index (kg/m^2^)	23.26 ± 4.04
Injured side	
Right/Left	9 (52.94)/8 (47.06)
Dominant side	
Right/Left	17 (100)/0 (0)
Time since first sprain	
3–6 months	5 (29.41)
6–12 months	1 (5.88)
1–5 years	9 (52.94)
5 years <	2 (11.76)
CAIT score	
Injured side/Intact side	8.06 ± 5.26/26.12 ± 4.74

Abbreviation: CAIT, Cumberland ankle instability tool.

### 5.1 Comparison of muscle architectures for injured and intact side

The PA values of the PL, MGCM, and TA in the MVC condition on the injured side were significantly different from those on the intact side (*p* < 0.05). There was only a significant difference in the PA of the MGCM in the resting condition between the injured and intact side (*p* < 0.05). In contrast, the MT of all muscles on the injured side were not significantly different in the resting and MVC conditions compared to the intact side (Table 2).

**TABLE 2:** Ultrasound‐based assessment results of muscle architectures (*N* = 17).

Variables	Injured side	Intact side	*t* (*p‐*value)
PA (°)			
PL			
Rest	8.02 ± 2.78	9.04 ± 2.20	−1.826 (0.087)
MVC	8.55 ± 1.77	11.52 ± 2.89	**−5.151 (<0.001)**
PB			
Rest	9.87 ± 2.29	11.57 ± 3.00	−2.082 (0.054)
MVC	11.37 ± 3.69	12.02 ± 3.64	−1.081 (0.296)
MGCM			
Rest	18.24 ± 2.94	20.73 ± 3.03	**−3.333 (0.004)**
MVC	32.76 ± 8.11	39.91 ± 6.13	**−4.667 (<0.001)**
TA			
Rest	7.60 ± 3.09	8.15 ± 2.09	−0.836 (0.416)
MVC	10.16 ± 4.10	12.86 ± 4.22	**−2.918 (0.010)**
MT (cm)			
PL			
Rest	0.70 ± 0.24	0.74 ± 0.20	−0.989 (0.337)
MVC	0.64 ± 0.19	0.70 ± 0.21	−1.354 (0.194)
PB			
Rest	1.14 ± 0.33	1.20 ± 0.29	−1.236 (0.234)
MVC	1.01 ± 0.23	1.07 ± 0.22	−1.775 (0.095)
MGCM			
Rest	1.63 ± 0.35	1.72 ± 0.32	−1.775 (0.095)
MVC	1.54 ± 0.33	1.54 ± 0.21	−0.132 (0.897)
TA			
Rest	2.45 ± 0.55	2.47 ± 0.54	−0.342 (0.737)
MVC	2.68 ± 0.50	2.65 ± 0.56	0.418 (0.682)

*Note*: *p*‐values less than 0.05 in bold to indicate their statistical significance.

Abbreviations: MGCM, medial gastrocnemius; MT, muscle thickness; MVC, maximal voluntary contraction; PA, pennation angle; PB, peroneus brevis; PL, peroneus longus; TA, tibialis anterior.

*p* < 0.05.

### 5.2 Comparison of muscle peak torque (Nm/kg) between injured and intact side, and deficit ratio

As shown in Table 3, compared with those on the intact side, all normalized peak torques on the injured side were significantly lower in each direction at both velocities (*p* < 0.05).

**TABLE 3:** Isokinetic test results of peak torque and deficit (*N* = 17).

Peak torque (nm/kg)	Injured side	Intact side	Deficit (%)	*t (p*‐value)
AE				
30°/s	0.32 ± 0.10	0.38 ± 0.13	14.39 ± 13.57	**−4.245 (0.001)**
120°/s	0.22 ± 0.09	0.27 ± 0.09	19.01 ± 19.13	**−3.986 (0.001)**
AI				
30°/s	0.30 ± 0.09	0.39 ± 0.15	21.11 ± 21.25	**−3.508 (0.003)**
120°/s	0.21 ± 0.07	0.26 ± 0.08	14.99 ± 29.19	**−3.093 (0.007)**
AP				
30°/s	0.98 ± 0.44	1.40 ± 0.54	28.79 ± 18.43	**−5.649 (<.001)**
120°/s	0.61 ± 0.35	0.92 ± 0.41	33.57 ± 23.59	**−4.953 (<.001)**
AD				
30°/s	0.38 ± 0.11	0.49 ± 0.14	21.94 ± 11.85	**−6.201 (<.001)**
120°/s	0.19 ± 0.07	0.26 ± 0.09	26.90 ± 21.92	**−4.977 (<.001)**

*Note*: *p*‐values less than 0.05 in bold to indicate their statistical significance.

Abbreviations: AD, ankle dorsiflexion; AE, ankle eversion; AI, ankle inversion; AP, ankle plantar flexion.

*p* < 0.05.

### 5.3 Correlation between the relative PA ratio (compared to the contralateral side) in MVC and relative peak torque (compared to the contralateral side)

The results of the correlation analysis between the PA and isokinetic peak torque values are presented in Table 4. The relative isokinetic peak torque ratio of AD at 30°/s showed significant moderate correlations with the relative PA ratio of the TA in the MVC (*p* < 0.05; *r* = 0.498). No significant correlations were observed between other variables.

**TABLE 4:** Pearson correlation coefficients between relative PA ratio at MVC and relative peak torque ratio (*N* = 17).

Variables		
Relative PA ratio at MVC – relative peak torque ratio	Pearson *r* (*p‐*value)	95% CI
PL– AE 30°/s	−0.213 (0.412)	−0.629–0.298
PL– AE 120°/s	−0.207 (0.426)	−0.625–0.304
PB– AE 30°/s	0.225 (0.385)	−0.286–0.637
PB– AE 120°/s	−0.129 (0.621)	−0.574–0.375
MGCM– AP 30°/s	0.098 (0.708)	−0.401–0.553
MGCM– AP 120°/s	−0.072 (0.783)	−0.534–0.423
TA– AD 30°/s	**0.491 (0.045)**	0.014–0.786
TA– AD 120°/s	0.194 (0.456)	−0.441–0.518

*Note*: *p*‐values less than 0.05 in bold to indicate their statistical significance.

Abbreviations: AD, ankle dorsiflexion; AE, ankle eversion; AP, ankle plantar flexion; CI, confidence interval; MGCM, medial gastrocnemius; MVC, maximal voluntary contraction; PA, pennation angle; PB, peroneus brevis; PL, peroneus longus; TA, tibialis anterior.

*p* < 0.05.

## 6 DISCUSSION

The primary findings of this study were as follows: (1) PA of the MGCM in the resting condition and PA of the TA, MGCM, and PL in the MVC condition were significantly reduced on the injured side compared to the intact side; and (2) significant differences were observed in muscle strength for both limbs in all four directions at 30°/s and 120°/s. A secondary finding was that PA (ratio of injured side to intact side in MVC condition) of the TA showed a moderate correlation with dorsiflexion (peak torque ratio of the injured side to intact side) at 30°/s.

This is the first study to quantify changes in architectural parameters (MT and PA) and strength of the peri‐ankle muscles bilaterally under resting and MVC conditions in patients with CAI. Understanding the changes of architectures and performance of the peri‐ankle muscles will provide opportunities to better understand how injuries in patients with CAI may be related to changes in ankle and foot function.

Yoshida and Suzuki (2020) evaluated the PA and MT of the peroneal and GCM muscles during a single‐leg standing task in patients with CAI [26]. They found significant differences in the PA of the PL between the injured and intact side. Although the PA in the GCM group was not statistically significant, this may be due to the different posture compared to the one used in our study for measuring MVC. In future research, comparative values analyzed in a consistent posture will be required. PA refers to the arrangement of muscle fibers in relation to the axis of force generation. It is known to play a significant role in determining muscle performance [27]. In particular, the MGCM showed significant changes in the PA under the two conditions (rest and MVC) in both ankles in our study. These muscles are generally larger than other muscles in the calf and are prime movers in AP; thus, their architecture is related to force/power production. However, decreased muscle activation of the injured side results in fewer fascicle insertions being pulled together, in turn causing less pinnation change and thus a smaller overall PA [28, 29]. Therefore, it is thought that the MGCM showed a clear difference, unlike the other muscles (peroneus muscles and TA).

MT is a known factor of muscle strength and has been used as an indicator of muscle weakness and atrophy [30]. Interestingly, we did not identify any differences in the MT between the injured and intact side, which is consistent with the findings of Abdeen et al. (2019) who studies measuring the MT of the peroneus muscle [31]. Similarly, Tashiro et al. (2021) found no differences between the CAI and healthy groups in the MT of the PL and TA [32]. When muscle use is restricted, loss of muscle strength is common in proportion to muscle size reduction [33]; however, the degree of muscle atrophy and weakness may vary depending on physical activity [34]. This suggests that the present and previously reported features of patients with CAI may be related to PA rather than MT.

We identified large deficits in concurrent 4‐way ankle strength weakness in patients with CAI. The results were consistent with previous studies (CAIT score = 18.5 ± 4.7) [32]. Although ligaments provide static restraint and help maintain the bony integrity of the joint, the peri‐ankle muscles must perform effectively to provide crucial active joint stability [35]. The asymmetrical strength of muscle groups within the leg is linked to the risk of injury [36]; therefore, it is important to understand changes in muscle architecture and function to minimize the risk of injury.

The moderate correlation observed between the PA of the relative TA (compared to the intact side) at the MVC and the peak torque of relative dorsiflexion at 30°/s (compared to the intact side) is novel, and these measurements have not been previously documented in existing literature. Although we did not observe a relationship between PA and muscle function in other muscles (peroneus muscles and MGCM), our findings are significant due to the important role of the TA, which is a crucial part of the dynamic defense mechanism responsible for dorsiflexion in relation to injury. These findings may aid in the potential evaluation of force‐generating attenuation and AD movement in PA with respect to force‐generating principles and will help assess the effectiveness of interventions. However, the confidence interval of the isokinetic peak torque correlation data in TA was in the range of 0.014–0.786, which weakens the clinical significance of our findings. Additional research is necessary to further investigate this relationship.

The study has several limitations that would be considered when interpreting its results. Firstly, the sample size was small and skewed toward female patients, with a focus on severe cases of chronic instability seen at tertiary hospitals. This means that it may not be appropriate to generalize the findings to the broader patients with CAI. Future research suggests considering factors such as gender, duration of chronic instability, subjective symptoms, activity level, and previous treatments to provide a more comprehensive understanding. Secondly, as the research was conducted retrospectively and only a single image was assessed for each muscle, there was possibility of measurement error. However, the measurement tools and procedures used in this study have been proven reliable and valid in prior studies [17].

## 7 CONCLUSION

The study showed that patients suffering from long‐term chronic instability following an ankle sprain not only experience reduced muscle performance around the ankle but also have changes in the architecture of their muscles, particularly in the PA. The study also confirmed a relationship between the decline in TA muscle performance and the changes in architecture. These results can help improve the initial diagnosis and management of muscle conditions for patients with CAI.

## AUTHOR CONTRIBUTIONS

**Heeju Yu**: Conceptualization (lead); writing – original draft (lead); formal analysis (lead); investigation (equal); methodology (equal); writing – review & editing (equal). **Seungmi Yeo**: Conceptualization (supporting); writing – original draft (supporting); investigation (equal); writing – review & editing (equal). **Ji Young Lim**: Conceptualization (supporting); writing – original draft (supporting); formal analysis (supporting); methodology (equal); writing – review & editing (equal). **Inah Kim**: Investigation (equal); writing – review and editing (equal). **Jihye Hwang and Wan‐hee Lee**: Project administration (lead); supervision (lead); conceptualization (supporting); writing – review & editing (equal); methodology (equal).

## CONFLICT OF INTEREST STATEMENT

The authors declare no conflicts of interest.

## ETHICS STATEMENT

Ethical approval for this retrospective study was provided from the Samsung Medical Center Institutional Review Board (IRB No. 2022‐11‐086).

## Data Availability

The datasets used and/or analyzed in the current study are available from the corresponding author upon reasonable request.
